# Genetic and Neurodevelopmental Markers in Schizophrenia-Spectrum Disorders: Analysis of the Combined Role of the *CNR1* Gene and Dermatoglyphics

**DOI:** 10.3390/biomedicines12102270

**Published:** 2024-10-07

**Authors:** Maria Guardiola-Ripoll, Alejandro Sotero-Moreno, Boris Chaumette, Oussama Kebir, Noemí Hostalet, Carmen Almodóvar-Payá, Mónica Moreira, Maria Giralt-López, Marie-Odile Krebs, Mar Fatjó-Vilas

**Affiliations:** 1 FIDMAG Germanes Hospitalàries Research Foundation, 08830 Sant Boi de Llobregat, Spain; 2 CIBERER (Biomedical Research Network in Rare Diseases), Instituto de Salud Carlos III, 28029 Madrid, Spain; 3 CIBERSAM (Biomedical Research Network in Mental Health), Instituto de Salud Carlos III, 28029 Madrid, Spain; 4 Departament de Biologia Evolutiva, Ecologia i Ciències Ambientals, Facultat de Biologia, Universitat de Barcelona, 08028 Barcelona, Spain; 5 Université Paris Cité, Institute of Psychiatry and Neuroscience of Paris (INSERM U1266), GHU-Paris Psychiatrie et Neurosciences, 75014 Paris, France; 6 Department of Psychiatry, McGill University, Montreal, QC H3A 0G4, Canada; 7 Servei de Psiquiatria Infantil i de l’Adolescència, Hospital Universitari Germans Trias i Pujol, 08916 Badalona, Spain; 8 Departament de Psiquiatria i Medicina Legal, Universitat Autònoma de Barcelona (UAB), 08193 Cerdanyola del Vallès, Spain

**Keywords:** schizophrenia-spectrum disorders, endocannabinoid system, *CNR1*, dermatoglyphics, neurodevelopmental biomarkers

## Abstract

**Background:** Dermatoglyphic pattern deviances have been associated with schizophrenia-spectrum disorders (SSD) and are considered neurodevelopment vulnerability markers based on the shared ectodermal origin of the epidermis and the central nervous system. The endocannabinoid system participates in epidermal differentiation, is sensitive to prenatal insults and is associated with SSD. **Objective:** We aimed to investigate whether the Cannabinoid Receptor 1 gene (*CNR1*) modulates the dermatoglyphics–SSD association. **Methods:** In a sample of 112 controls and 97 patients with SSD, three dermatoglyphic markers were assessed: the total palmar a-b ridge count (TABRC), the a-b ridge count fluctuating asymmetry (ABRC-FA), and the pattern intensity index (PII). Two *CNR1* polymorphisms were genotyped: rs2023239-T/C and rs806379-A/T. We tested: (i) the *CNR1* association with SSD and dermatoglyphic variability within groups; and (ii) the *CNR1* × dermatoglyphic measures interaction on SSD susceptibility. **Results:** Both polymorphisms were associated with SSD. The polymorphism rs2023239 modulated the relationship between PII and SSD: a high PII score was associated with a lower SSD risk within C-allele carriers and a higher SSD risk within TT-homozygotes. This result indicates an inverse relationship between the PII and the SSD predicted probability conditional to the rs2023239 genotype. **Conclusions:** These novel findings suggest the endocannabinoid system’s role in the development and variability of dermatoglyphic patterns. The identified interaction encourages combining genetic and dermatoglyphics to assess neurodevelopmental alterations predisposing to SSD in future studies.

## 1. Introduction

Schizophrenia-spectrum disorders (SSD) encompass different severe mental disorders, including schizophrenia, schizoaffective and schizophreniform disorders. The central etiological hypothesis sustains that SSD emerge as the consequence of multiple genetic and environmental factors altering the homeostasis of neurodevelopmental trajectories during the intrauterine and early postnatal periods, as well as during childhood and early adolescence [1,2]. This model is supported by the higher prevalence observed in patients as compared to healthy controls of different indirect markers of neurodevelopment disturbances, such as minor physical anomalies [3,4] or neurological soft signs [5], which have been described alongside neuroanatomical [6,7] and neurofunctional changes [8,9].

From this view, other ectodermal tissue derivatives, such as dermatoglyphics, have captured much attention as early intrauterine neurodevelopmental markers [10,11,12]. The dermatoglyphic patterns are grooved configurations on palms and soles’ surfaces conformed by the alternation of epidermal ridges and sulci. These patterns are established from the 6th to the 24th week of gestation when their formation is complete, and they remain unchanged over the lifetime [10,11]. This process occurs in parallel with several crucial central nervous system development processes, such as neural proliferation, cortex migration, and prosencephalic development [13,14,15]. Thus, dermatoglyphics represent evidence of a particular neurodevelopmental window, and dermatoglyphic alterations may be informative about early deviances in this process. The consideration of dermatoglyphics as indirect markers of neurodevelopmental alterations is supported by the high occurrence of dermatoglyphic deviations in chromosomal syndromes and neurodevelopment-related disorders caused by genetic and environmental factors [16]. Considering the neurodevelopmental roots of SSD, several studies have reported quantitative and qualitative dermatoglyphic differences between patients affected by these disorders and healthy controls. Generally, patients tend to present simplified dermatoglyphic configurations and higher bilateral asymmetry [17,18,19]. Indeed, dermatoglyphic pattern deviances have been highlighted as a relevant schizophrenia risk factor through an umbrella review [20].

Family and twin-based studies have determined dermatoglyphic heritability in variable but significant levels (h^2^ = 0.65 to 0.96) [16,21,22]. While little is known about dermatoglyphics-specific genetic determinants, a recent GWAS identified 18 loci associated with fingerprint type that highlighted the role of pathways related to limb development [23]. Nonetheless, information as to which extent the dermatoglyphics’ genetic and environmental determinants are shared with those of SSD is still missing. Among the mechanisms proposed to mediate gene and environment interactions on SSD and dermatoglyphic morphology, prenatal stress, and obstetric complications achieve importance [24,25,26]. In this sense, several studies have shown a link between obstetric complications and dermatoglyphic ridge count reductions in schizophrenia [12,19]. Then, fetal hypoxia seems to be a common denominator in the obstetric adverse events associated with psychosis based on gene–environment interaction studies and meta-analyses [25,27].

Oxygen level variation is required for several physiological processes to take place, such as the formation of the neural fold [28], the neural tube closure [29] and oligodendrocyte proliferation and myelination [30]. Accordingly, the regulation of hypoxia and its molecular mechanisms are considered essential for neurodevelopment and dysfunctions in such homeostasis may lead to abnormal gene expression and lasting changes in neuronal circuitry in the developing brain [31]. Interestingly, several reviews highlighted that, among schizophrenia’s candidate genes, more than half of them met the criteria for a link to ischemia-hypoxia and/or vascular factors [32,33].

Among the genes highlighted in Schmidt–Kastner’s studies [32,33], there is the Cannabinoid Receptor 1 gene (*CNR1)*. An increasing body of evidence has emphasized the role of *CNR1* in the genetic underpinnings associated with schizophrenia [34]. It has been suggested that *CNR1* mediates the relationship between environmental risk factors and changes in brain structure and cognitive function in schizophrenia [35]. The *CNR1* encodes for the CB1 receptor, the main endocannabinoid receptor in the brain and an essential central nervous system presynaptic receptor [36,37,38]. Additionally, the endocannabinoid system plays a key role in the modulation of the dopaminergic neurotransmission system [39]. Indeed, dopaminergic dysregulation has been largely associated with the presence of psychotic symptoms [40]. The fact that exogenous cannabinoids also impact the synthesis and release of dopamine [41] indicates not only the endocannabinoid–dopaminergic interaction, but also the importance of these synergies for the understanding of the etiology of SSD and the response to different antipsychotic agents.

Even though the endocannabinoid system’s role in epidermal differentiation has been barely studied, it is known that the CB1 receptor modulates human keratinocytes, epidermal differentiation and skin development [42]. It has also been described involved in the epidermal permeability barrier [43]. Such particular functions of CB1 are integrated into the well-described roles of the endocannabinoid system in the regulation of cell-fate processes during development, including cell survival, proliferation, differentiation, and migration [44,45,46]. The CB1 receptors are expressed during early development in neuroepithelial progenitor cells [44] and have been detected in the fetal brain as early as week 14 in regions of the frontal cortex, hippocampus, caudate nucleus, putamen, and cerebellum, mimicking their adult brain detection [47,48,49]. In addition, the *CNR1* mRNA shows evidence of upregulation during ischemia [50], emphasizing its expression sensitivity to oxygen levels.

Considering the shared genetic background between the development of the brain and dermatoglyphics and the role of the endocannabinoid system during these processes, we hypothesized that *CNR1* genetic variability influences both the dermatoglyphic configurations and the liability towards SSD and that it also modulates the relationship between dermatoglyphic pattern deviances and SSD risk. We aimed to investigate the common underpinnings of SSD and dermatoglyphic patterns and combine phenomics of the dermatoglyphic patterns with the *CNR1* genetic data to identify specific biomarkers for characterizing the liability toward SSD. Particularly, we examined: i) the genetic association of two *CNR1* polymorphisms with SSD, ii) the impact of the *CNR1* variability on various dermatoglyphic measures; and iii) whether *CNR1* genetic variants modulate the relationship between the dermatoglyphic variables and susceptibility to SSD.

## 2. Materials and Methods

### 2.1. Sample

The study sample consisted of 209 unrelated French Caucasian individuals, including 112 healthy controls (HC) and 97 individuals diagnosed with a SSD. All patients met the DSM-IV criteria for SSD, confirmed through the Diagnostic Interview for Genetic Studies (DIGS version 3.0) [51]: 82.5% schizophrenia, 15.5% schizoaffective disorder, and 2.0% schizophreniform disorder. HC participants were recruited via local advertisements and screened with DIGS 3.0 based on the following criteria: Caucasian ancestry, no family history of schizophrenia, alcoholism, or bipolar disorder in first- or second-degree relatives, and no personal history of DSM-IV Axis I mental disorders. Exclusion criteria for both groups included chromosomal syndromes. Between controls and patients, there were differences regarding sex distribution (41 HC males (36.6%) and 69 SSD males (71.1%); χ^2^ = 24.86, *p* < 0.001) and age (HC = 25.85 (SD = 6.54) and SSD = 29.20 (SD = 7.75); U = 3624.5 *p* < 0.001).

### 2.2. Genotyping

All the individuals were genotyped for two SNPs in the *CNR1* gene (6q15): the rs2023239-T/C and the rs806379-A/T. These SNPs were selected based on their minor allele frequency in European populations (>5%), their potential relevance in relation to protein availability, and their role in the pathophysiological mechanisms associated with SSD [52,53]. Genomic DNA was extracted from peripheral blood cells by means of precipitation using Genisol Maxi-Prep Kit (ABgene, Epsom, UK), and the selected variants were genotyped using a fluorescence-based allelic discrimination assay (TaqMan, Applied Biosystems, Foster City, CA, USA), with standard conditions.

### 2.3. Dermatoglyphics Assessment

Bilateral finger and handprints were obtained from all participants using Speedball Block Printing Inks (Speedball Block Ink, Utrecht Art Supplies, Cranbury, NJ, USA) by engraving whole-hand as well as each fingerprint’s impressions on a white paper surface. The use of a magnifying lens and digitalized images of all prints allowed finger figure identification and palmar ridge counts.

**Figure 1:**
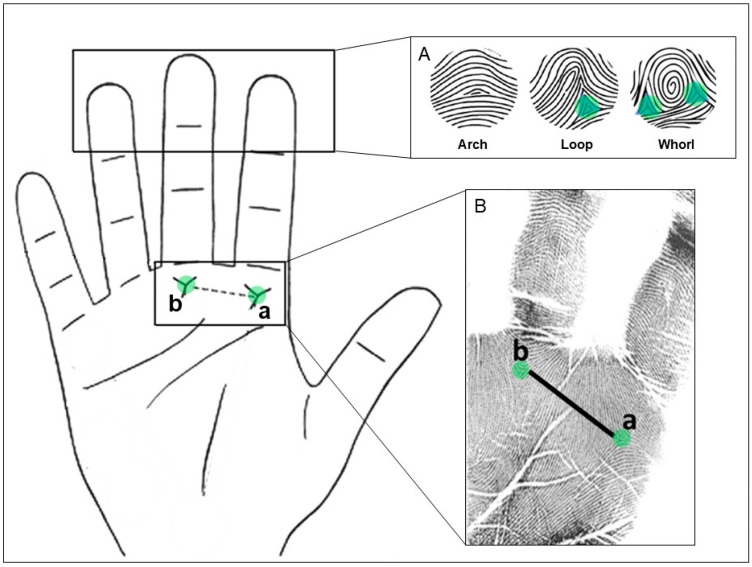
(**A**) Different fingertip patterns (left to right): arch, loop and whorl with the triradius marked with green circles. (**B**) Handprints where the triradii a and b are indicated. The total a-b ridge count (TABRC) corresponds to the sum of the number of ridges between both triradii from the right and left hands. Figures adapted from [12,23].

On each finger, we identified the fingertip pattern based on the number of triradii associated with each figure. A triradius is a Y-shaped point of convergence of ridges from 3 different directions. Then, the types of figures identified were arches (with zero triradii), loops (with one triradius) and whorls/double-loops (with two triradii) (Figure 1A). After, we calculated the pattern intensity index (PII) by adding up the total number of triradii and dividing the sum by the number of fingers analyzed. This quantifies the number of triradii in the ten fingers and measures the complexity of the finger configurations [16].

On the palms, we analyzed the a-b ridge count (ABRC) of both hands, which measures the size of the second interdigital area of the hand located between the bases of the index and medium fingers. This is made by counting the number of ridges between the triradius a (in the base of the index finger) and the triradius b (in the base of the medium finger) (Figure 1B). Then, we computed (i) the total a-b ridge count (TABRC), which is the addition of the right and the left ABRC; and (ii) the a-b ridge count fluctuating asymmetry (ABRC-FA), a measure of developmental instability, which is the absolute difference between the right and the left ABRC.

The final number of individuals analyzed for each dermatoglyphic variable varies depending on the quality of the fingerprints. Dermatoglyphic variables assessment was performed according to Cummins and Midlo [54], by one of the authors (M.F-V.) who was blind to the status of the subjects, and in the same way as described in [12].

### 2.4. Statistical Analyses

All the data was processed in SPSS (SPSS 27.0, IBM SPSS Statistics for Windows, version 27.0, released 2020, IBM Corporation, Armonk, NY, USA). The Hardy–Weinberg equilibrium of the genotypes and the genetic models was tested using PLINK v1.07 [55], a toolset designed to develop genetic association analysis in a computationally efficient manner. Tests for sex distribution and age differences across diagnostic categories were conducted using chi-square (χ^2^) and Mann–Whitney (U) tests, respectively (SPSS).

Based on the sample distribution and to maximize the power, all the analyses were conducted assuming a minor allele dominance model. Then, the genotypes were dichotomized by grouping the minor and the heterozygous genotypes (rs2023239-TC/CC (C-allele carriers (Ccar)) vs. rs2023239-TT, and rs806379-AT/TT (T-allele carriers (Tcar)) vs. rs806379-AA).

Firstly, we examined the genetic association of *CNR1*-rs2023239 and *CNR1*-rs806379 genotypes with the risk for SSD. Secondly, we evaluated the effect of the dominant model on each dermatoglyphic measure separately in each diagnostic group. Lastly, we explored whether there was a modulation effect of the *CNR1* variants on the relationship between dermatoglyphic variables and SSD vulnerability. For this purpose, we tested the interaction between the genotypes and each of the dermatoglyphic measures on the risk for SSD. All the analyses were conducted with logistic or linear regressions, when appropriate, and included sex as a covariate. When the nominal *p*-values (p_nom_) reached the significance threshold (p_nom_ ≤ 0.05), the empirical *p*-values (p_emp_) obtained after a 10,000 permutations procedure are reported, with a significance threshold set at p_emp_ ≤ 0.05. To comprehend the effect of the significant interactions detected with PLINK, we subsequently obtained the corresponding predicted probabilities and plotted them (SPSS).

We calculated the genetic power of our case-control sample using the Quanto v1.2.4 [56] by assuming an additive model, a disease prevalence of 3% and the minor allele frequencies observed in our sample. The two markers had an 80% power to detect a genetic effect with an OR  ≥  1.48. For the post hoc statistical power calculation of the association analyses between the polymorphisms and dermatoglyphic variables, we used G*Power 3.1.9 [57]. As regards the rs2023239, our sample was powered (1-β = 0.80, α = 0.05) to detect intermediate effect sizes (d > 0.52, both in HC and SSD) in the between-groups comparison of the dermatoglyphic variables. For example, it corresponds to a difference of 5.85 in the TABRC or 0.19 in the PII between the TT genotype and Ccar. Concerning the rs806379, our sample was powered to detect intermediate effect sizes (HC: d > 0.48; SSD: d > 0.56), which represents a difference of 5.95 in the TABRC or 0.20 in the PII between the AA genotype and Tcar.

## 3. Results

### 3.1. Dermatoglyphic Assessment

The dermatoglyphic data of the variables used in the analysis (TABRC, ABRC-FA, and PII) are reported in Table 1 and Table 2. As shown in these tables, there were no dermatoglyphic differences between males and females in the whole sample or within diagnostic groups (Table 1). Within patients, we did not observe any difference between left and right ABRC or PII, while controls did present larger left-hand ABRC, but similar PII scores (Table 2). Further data on finger figure frequencies are given in Supplementary Table S1.

### 3.2. Case-Control Genetic Association Analyses

The minor alleles identified in our whole sample matched those described for the European Population from the 1000 Genomes (rs2023239-C-allele and rs806379-T-allele). The minor allele frequencies in our whole sample (HC and patients) were 0.359 (rs2023239 C-allele) and 0.352 (rs806379 T-allele), showing some difference from those in the 1000 Genomes European Population (0.157 and 0.446, respectively) (the detailed group frequencies are displayed in Table 3). Despite these differences, the Hardy–Weinberg equilibrium analyses conducted with PLINK indicate that the observed genotype frequencies do not depart from the expected, fulfilling the equilibrium both in the whole sample (rs2023239 p_nom_ = 0.13 and rs806379 p_nom_ = 0.23) and within each group (HC: rs2023239 p_nom_ = 0.70 and rs806379 p_nom_ = 0.45; Patients: rs2023239 p_nom_ = 0.42 and rs806379 p_nom_ = 1). Neither allele nor genotype frequencies differed between males and females.

Genetic association analysis showed that both *CNR1* polymorphisms were significantly associated with the disorders’ liability (Table 3). Regarding rs2023239, we detected an overrepresentation of Ccar among HC compared to patients; therefore, the TC/CC genotypes were associated with a protective effect (OR < 1). Concerning rs806379, we detected an overrepresentation of Tcar among patients with SSD; then, the AT/TT genotypes were associated with a risk effect (OR > 1).

### 3.3. CNR1 Genotypes Effect on Dermatoglyphic Patterns

We inspected the genotypic effect on TABRC, ABRC-FA, and PII within each diagnostic group (Table 4).

The data revealed effects within HC. First, the rs2023239-Ccar presented higher PII scores as compared to TT-homozygous (β = 0.354, se = 0.140, 95%CI = 0.081:0.628, W = 2.537, p_nom_ = 0.017, p_emp_ = 0.016. Second, the rs806379-Tcar had lower TABRC as compared to AA-homozygous (β = −4.370, se = 2.089, 95%CI = −8.464:−0.276, W = −2.092, p_nom_ = 0.039, p_emp_ = 0.038. No *CNR1* effect on dermatoglyphic markers was detected in patients.

**Figure 2:**
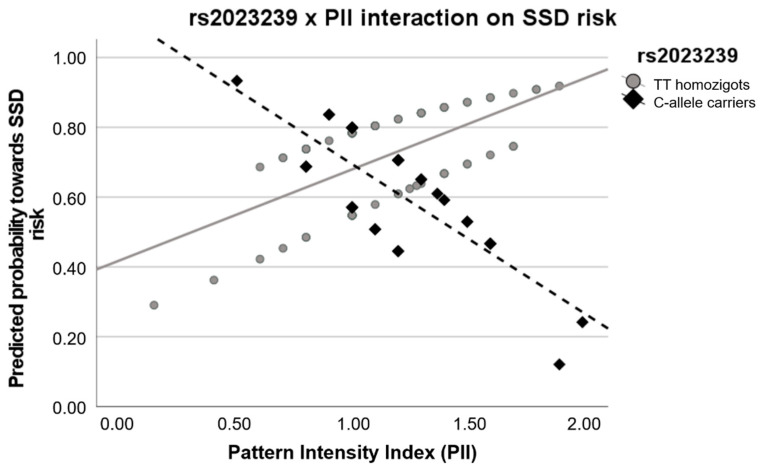
Interaction graph showing the interplay between the *CNR1*-rs2023239 genotype and the pattern intensity index (PII) on the risk for schizophrenia-spectrum disorders (SSD). A lower predicted probability indicates a lower risk towards SSD. Values show the inverse relationship between PII and predicted probability for SSD depending on the rs2023239 genotype.

Lastly, we found that *CNR1*-rs2023239 variability significantly modulated the relationship between the PII and SSD risk. The logistic regression model (including the rs2023239, the PII and their interaction) was globally significant (W = 9.822, p_nom_ = 0.044, p_emp_ = 0.044), as well as the interaction term (W = −2.361, se = 1.604, OR = 0.023, OR [95%CI] = 0.001:0.526, p_nom_ = 0.018, p_emp_ = 0.039). Subsequently, the predicted probabilities were obtained and plotted (Figure 2). The scatter plot showed that the relationship between the SSD risk and the PII was inverse depending on the rs2023239 genotype. More specifically, the individuals who were rs2023209-Ccar and had a higher PII presented a lower predicted probability towards SSD risk. In contrast, AA-homozygous and the same high levels of PII depicted the opposite relationship with the risk.

## 4. Discussion

This study aimed to investigate the shared genetic underpinnings of SSD and dermatoglyphic patterns by assessing whether *CNR1* genetic variability influences the relationship between dermatoglyphic pattern deviances and SSD liability. To the best of our knowledge, this is the first study to explore the role of the endocannabinoid system in dermatoglyphic pattern variability, and the derived results point towards the combined effect of *CNR1* and dermatoglyphics on modulating the risk towards SSD.

First, our results suggest that the two *CNR1* polymorphisms (rs2023239 and rs806379) may play a role in predisposing individuals to SSD. As reviewed by Gouvêa et al. [34], although numerous association studies have explored the impact of *CNR1* variability on schizophrenia, SSD, and other related clinical outcomes with largely negative results, the existing data is quite heterogeneous in terms of population origin and SNP selection. Regarding our sample, particularly in the HC group, the observed allelic frequencies differ from those reported in the European population of the 1000 Genomes Project. Several factors may account for this discrepancy. First, the 1000 Genomes data represents the broader European population, while our sample is exclusively of French origin, which may introduce specific population variations. Second, the limited size of our sample could also be related to certain hazardous deviations of the observed allelic frequencies in the reference population. However, such potential bias may be mitigated by the fact that the genotypic frequencies in our whole sample and in each group separately fulfill the Hardy–Weinberg equilibrium. However, sampling bias is a potential factor that can occur in studies with limited sample sizes, and therefore, results should be interpreted carefully, especially when extrapolating our findings to other populations. Therefore, as emphasized by the above-mentioned review, new investigations on the *CNR1* role in the risk for psychiatric disorders are needed. 

Focusing on the rs2023239, while there is no previous evidence of its association with the risk for psychosis per se, a modulation effect in the evolution of the psychopathological features and brain structural changes along the course of first-episode psychosis has been described [34,58]. This polymorphism has also been associated with the risk of metabolic syndrome in patients with SSD [59]. On the other hand, previous studies failed to associate the rs806379 with the risk for schizophrenia in a Brazilian sample [34], or with the risk for metabolic syndrome [59]. However, considering other phenotypes associated with psychosis, evidence indicates that, under exposure to early psychosocial adversity, the rs806379 modulates impulsivity control in healthy adolescents [60]. These associations may be driven by mechanisms unrelated to protein sequence since they lie in intronic regions, but they may be related to expression regulatory mechanisms. In this sense, the rs2023239 seems to influence CB1 receptor density in lymphocytes [52]. If lymphocyte CB1 levels mimic the central nervous system ones, receptor availability changes could result in neurotransmission effects. Indeed, these two SNPs (rs2023239 and rs806379) in combination with another (rs1535255) have been associated with low levels of CB1 receptor mRNA in the cerebral cortex and the midbrain [53], reinforcing the evidence of the modulatory effects of intronic variants on the receptor availability in the brain. It is also of interest that CB1 levels influence the expression of differentiation signals in various neuronal lineages [44] and that several studies report altered endocannabinoid receptor concentrations in patients with schizophrenia in the dorsolateral prefrontal cortex, the posterior and anterior cingulate cortex [49,61,62,63].

Second, our findings link the *CNR1* rs2023239 and rs806379 variants with PII and TABRC variability inHC. On the one hand, we describe that the rs2023239-C allele is associated with higher PII, which means that this allele, observed in less frequency in patients than in controls in our sample, is in turn related to higher dermatoglyphic complexity, as represented by the presence of whorls and loop patterns. These results align with previous data reporting lower finger dermatoglyphic patterns complexity assessed through the frequency of the fingertip figures in schizophrenia and schizotypal traits [64,65,66]. On the other hand, studies assessing TABRC as a developmental biomarker evidenced ridge count reductions in patients with schizophrenia and with SSD, as well as in subgroups of patients with reported perinatal complications [12,17,18,19]. Hence, the detected association of the rs806379-Tcar with TABRC reductions in HC would also support previous findings from case-control association studies

We would have expected also to find a *CNR1* modulation effect on patients’ dermatoglyphic measures. Nonetheless, these results could reflect, on the one hand, the low expected penetrance that two single common variants have on these complex phenotypic measures. On the other hand, considering a multifactorial and polygenic context, we must consider the effect of different genetic and environmental forces underlying the dermatoglyphic configurations, as in the risk for psychosis [12,19,67,68]. Therefore, by analyzing two SNPs, it is noticeable that we are focusing on a particular biological pathway to assess its effect on dermatoglyphic complexity and we should not forget that the developmental stability patterns of an individual are shaped by its global genetic makeup together with the environmental context [12,19]. Accordingly, our findings in HC and not in patients could indicate group differences in the sensitivity to gene–environmental insults along neurodevelopmental processes and, particularly, in the effects that such group-specific ontogenetic patterns may have on dermatoglyphic markers.

Third, we assessed whether *CNR1* modulated the dermatoglyphics SSD association. The analyses revealed an interplay between the rs2023239 and PII on the liability for these disorders. Individuals carrying the C-allele and with high PII scores showed a reduced liability for SSD, contrarily to TT-homozygous with the same PII. The interaction results are aligned with the case-control association data and the dermatoglyphic modulation effect observed within HC. On the one hand, these findings emphasize the modulation role of *CNR1* in the connection between dermatoglyphic markers and SSD, showing that the relationship between this neurodevelopmental marker and SSD liability can be inverse depending on the *CNR1* genotypes. The intricacy of these inverse genetic effects could be explained by the presence of differential adaptability, which allows risk and protective genotypes to persist through generations by potentially conferring adaptive effects to different environmental conditions. In the current framework, such conditions could be related to prenatal insults predisposing to SSD and having variable effects on the mechanisms involved in ectodermal derivatives development depending on the specific time of occurrence. On the other hand, our results relate the *CNR1* gene to a particular neurodevelopmental marker, the dermatoglyphic configurations. Many *CNR1* × environmental interactions have been described involving cannabis use, stressful life events and childhood adversity on SSD susceptibility, SSD brain-based phenotypes and other mental disorders [60,69,70]. This, together with evidence suggesting a hypoxia modulation effect on *CNR1* mRNA levels [50], could lead to thinking about an interplay between *CNR1* and adverse prenatal environmental factors impacting the developmental trajectories reflected in dermatoglyphic and brain alterations. In this sense, future studies extending our data by assessing obstetric and perinatal complications would be of great value to evaluate the environmental influences on the brain and dermatoglyphic variables and pave the way for gene × dermatoglyphics studies in neurodevelopmental disorders.

Lastly, some limitations should be acknowledged. First, new analyses in larger samples are needed to confirm our findings. Despite selecting two *CNR1* variants based on their relevance for SSD and neurodevelopment, the use of two SNPs neither represents the polygenic background of schizophrenia and SSD nor the whole genetic determinants of dermatoglyphic configurations. Further studies inspecting the role of genetic variants across the endocannabinoid system or even genome-wide on dermatoglyphic measurements captured through automated and multivariate approaches would help to comprehend the relationship between SSD and dermatoglyphics and to develop predictive statistical methodologies applied in the development of diagnostic tools. In this sense, the combination of genomic approaches with machine learning algorithms already developed based on dermatoglyphic patterns [71] will potentially enhance the development of tools that better identify vulnerable subgroups of patients with a higher burden of neurodevelopmental alterations. Moreover, since dermatoglyphic patterns can be easily accessed and assessed, they can become a very valuable asset for the development of these tools compared with more invasive, inaccessible, or overall expensive markers.

In conclusion, our results add to previous evidence implicating the endocannabinoid system with neurodevelopmental disorders, such as SSD, and represent new evidence regarding the *CNR1* gene in the development and variability of dermatoglyphic patterns. While these data are consistent with the established role of the Cannabinoid receptor 1 in epidermal differentiation and skin development, and its involvement in psychosis risk and environmental insults sensitivity, new research in larger samples is needed. These data open new venues for investigation by reflecting the complexity and multifactorial nature of both dermatoglyphic patterns and SSD and pointing towards the need to combine genomic and environmental data with different neurodevelopmental markers, not only to understand the etiological mechanisms of SSD but also to develop new tools to improve the characterization and treatment of individuals with a higher neurodevelopmental burden.

## Figures and Tables

**Table 1:** Dermatoglyphic variability by group and sex. Description of the dermatoglyphic variability between males (M) and females (F), and healthy controls (HC) and patients with schizophrenia-spectrum disorders (SSD) in the whole sample and separated by diagnosis.

Dermatoglyphic Variables		Whole Sample		HC	SSD
		Diagnosis	Sex		
TABRC	Mean (SD)	HC: 84.61 (10.41) SSD: 82.85 (10.86)	M: 86.22 (9.24) F: 83.68 (11.56)	M: 85.04 (9.33) F: 80.22 (11.76)	M: 87.78 (9.01) F: 84.62 (11.41)
	Statistics	β = −0.033=, se = 3.841, W = 3.841, p_nom_ = 0.050	T = 1.582 p_nom_ = 0.116	T = 1.744 p_nom_ = 0.086	T = 1.450 p_nom_ = 0.150
ABRC-FA	Mean (SD)	HC: 2.7 (1.96) SSD: 3.5 (3.53)	M: 3.24 (2.64) F: 3.70 (3.10)	M: 3.00 (2.81) F: 3.56 (3.19)	M: 3.57 (2.40) F: 3.74 (3.11)
	Statistics	β = −0.054, se = 0.062, W = 0.754, p_nom_ = 0.385	T = −1.038 p_nom_ = 0.301	T = −0.693 p_nom_ = 0.491	T = −0.296 p_nom_ = 0.768
PII	Mean (SD)	HC: 1.22 (0.32) SSD: 1.12 (0.32)	M: 1.19 (0.33) F: 1.10 (0.35)	M: 1.17 (0.03) F: 1.15 (0.29)	M: 1.26 (0.30) F: 1.03 (0.42)
	Statistics	β = 0.050, se = 0.652, W = 0.006, p_nom_ = 0.938	T = 1.339, p_nom_ = 0.184	T = 0.219 p_nom_ = 0.828	T = 1.752 p_nom_ = 0.090

Mean and standard deviation (sd) are reported for the total a-b ridge count (TABRC), the a-b ridge count fluctuating asymmetry (ABRC-FA), and the pattern intensity index (PII) in all groups. The statistical tests applied are either a T-test or a logistic regression covaried by sex, and the associated nominal *p*-values (p_nom_) are given.

**Table 2:** Dermatoglyphic variability by hand. Description of the dermatoglyphic variability between right (R) and left (L) hands in the whole sample and separated by diagnosis.

Dermatoglyphic Variables		Whole Sample	HC	SSD
ABRC	Mean (SD)	R: 41.70 (5.55) L: 44.14 (5.78)	R: 41.77 (5.86) L:43.29 (5.60)	R: 41.87 (6.33) L: 42.01 (5.10)
	Statistics	T= −3.186 p_nom_ = 0.002	T = −2.540 p_nom_ = 0.012	T = −0.154 p_nom_ = 0.878
PII	Mean (SD)	R: 1.16 (0.38) L: 1.11 (0.42)	R: 1.19 (0.35) L: 1.12 (0.37)	R: 1.20 (0.33) L: 1.13 (0.35)
	Statistics	T = 0.544 p_nom_ = 0.588	T = 1.294 p_nom_ = 0.197	T = 1.216 p_nom_ = 0.226

Mean and standard deviation (sd) are reported for the a-b ridge count (ABRC) and the pattern intensity index (PII) in healthy controls (HC) and patients with schizophrenia-spectrum disorders (SSD). T-test was applied and thus the T statistic and the associated nominal *p*-value (p_nom_) are given.

**Table 3:** Genetic association data of *CNR1* polymorphisms with Schizophrenia-Spectrum Disorders. Allelic and genotypic counts are given for the healthy controls (HC, n = 112) and patients with schizophrenia-spectrum disorders (SSD, n = 97).

**Alleles**	**HC**	**SSD**	**Statistics**	**Significance**
rs2023239 (T/C)	121/103 (0.54/0.46)	147/47 (0.76/0.24)	χ^2^ = 21.39 OR [95%CI] = 0.376 [0.245–0.572]	p_nom_ = 3.76 × 10^−6^ p_emp_ = 1.00 × 10^−4^
rs806379 (T/A)	167/57 (0.75/0.25)	104/90 (0.54/0.46)	χ^2^ = 20.00 OR [95%CI] = 2.535 [1.679–3.829]	p_nom_ = 7.73 × 10^−6^ p_emp_ = 1.00 × 10^−4^
**Genotypes**	**HC**	**SSD**	**Statistics**	**Significance**
Additive model rs2023239 (TT/TC/CC)	34/53/25 (0.31/0.47/0.22)	57/33/7 (0.59/0.34/0.07)	W = −4.182 OR [95%CI] = 0.377 [0.239–0.596]	p_nom_ = 2.89 × 10^−5^ p_emp_ = 1.00 × 10^−4^
Dominant model rs2023239 (TT vs. Ccar)	34/78 (0.31/0.69)	57/40 (0.59/0.41)	W = −3.995 OR [95%CI] = 0.285 [0.154–0.528]	p_nom_ = 6.46 × 10^−5^ p_emp_ = 2.00 × 10^−4^
Additive model rs806379 (AA/AT/TT)	64/39/9 (0.57/0.34/0.1)	28/48/21 (0.29/0.49/0.22)	W = 3.909 OR [95%CI] = 2.434 [1.558–3.803]	p_nom_ = 9.28 × 10^−5^ p_emp_ = 1.00 × 10^−4^
Dominant model rs806379 (AA vs. Tcar)	64/48 (0.57/0.35)	28/69 (0.29/0.71)	W = 3.768 OR [95%CI] = 3.249 [1.760–5.996]	p_nom_ = 1.65 × 10^−4^ p_emp_ = 4.00 × 10^−4^

The case-control statistical comparison parameters are shown: chi-squared/logistic regression statistics (χ^2^/W), Odds Ratio (OR) and confidence interval [95%CI], and both nominal (p_nom_) and empirical (p_emp_) *p*-values after permutation.

**Table 4:** Description of the dermatoglyphic traits according to group and genotype for the two *CNR1* variants.

SNP	Diagnosis	Genotype	TABRC	ABRC-FA	PII
rs2023239	HC	TT	82.86 (13.56)	3.14 (2.40)	1.03 (0.41)
		Ccar	86.84 (9.25)	3.88 (3.00)	1.29 (0.28)
	SSD	TT Ccar	83.70 (11.15)	2.97 (3.05)	1.19 (0.36)
			83.86 (8.93)	3.38 (2.72)	1.11 (0.33)
rs806379	HC	AA	87.49 (9.53)	3.90 (3.12)	1.18 (0.28)
		Tcar	83.43 (11.75)	3.39 (2.47)	1.12 (0.43)
	SSD	AA	85.94 (7.69)	3.06 (3.04)	1.23 (0.34)
		Tcar	82.94 (10.91)	3.18 (2.88)	1.14 (0.31)

HC: Healthy Controls; SSD: Schizophrenia Spectrum Disorder; TABRC: Total A-B Ridge Count; ABRC-FA: A-B Ridge Count–Fluctuating Asymmetry; PII: Pattern Intensity Index. Means (standard deviations) are given.

## Data Availability

The data supporting the findings of this study are available from the corresponding authors upon reasonable request.

## Supplementary Materials

The following supporting information can be downloaded at: https://www.mdpi.com/article/10.3390/biomedicines12102270/s1, Supplementary Table S1. Description of the finger figures frequency (%) between right (R) and left (L) hands in the whole sample, by diagnosis and by sex.
