# Supplementary material for: Genetic and Neurodevelopmental Markers in Schizophrenia-Spectrum Disorders: Analysis of the Combined Role of the CNR1 Gene and Dermatoglyphics
Source: Biomedicines. 2024 Oct 7;12(10):2270. doi: 10.3390/biomedicines12102270 (PMC11505170; doi:10.3390/biomedicines12102270)
Supplement: Supplementary file 1 [file biomedicines-12-02270-s001.zip › biomedicines-3019328-supplementary.pdf]

Supplementary Table S1. Description of the finger figures frequency (%) between right (R) and left (L) hands in the whole sample, by diagnosis and by sex. The figures are grouped by the number of triradii: arches with no triradii; loops with one triradius and whorl/double loops with two triradii. Chi-squared test (χ^2) was applied to assess for differences, and thus, the statistic and the associated nominal p-value (p_{nom}) are given.

		FINGER FIGURES FREQUENCY			
		**Arches**	**Loops**	**Whorls/Double loops**	**Statistics**
**ALL SAMPLE**	Right	0.052	0.703	0.246	χ^2=5.36 p_{nom} =0.069
	Left	0.084	0.708	0.208	
**HC**	Right	0.057	0.709	0.234	χ^2=1.730 p_{nom} =0.421
	Left	0.094	0.698	0.208	
**SSD**	Right	0.050	0.700	0.251	χ^2=3.72 p_{nom} =0.156
	Left	0.079	0.713	0.208	
**FEMALES**	Whole sample	R:0.052<br>L: 0.115	R:0.762<br>L:0.696	R:0.187<br>L:0.188	χ^2=5.19 p_{nom} =0.075
	HC	R:0.084<br>L:0.155	R:0.771<br>L:0.667	R:0.145<br>L:0.179	χ^2=2.66 p_{nom} =0.264
	SSD	R:0.027<br>L:0.084	R:0.755<br>L:0.720	R:0.218<br>L:0.196	χ^2=3.38 p_{nom} =0.184
**MALES**	Whole sample	R:0.052<br>L:0.065	R:0.666<br>L:0.715	R:0.282<br>L:0.220	χ^2=3.37 p_{nom} =0.185
	HC	R:0.027<br>L:0.027	R:0.640<br>L:0.733	R:0.333<br>L:0.240	χ^2=1.62 p_{nom} =0.446
	SSD	R:0.060<br>L:0.077	R:0.674<br>L:0.709	R:0.266<br>L:0.214	χ^2=2.03 p_{nom} =0.362
